# Bioactive Compounds of Agro-Industrial By-Products: Current Trends, Recovery, and Possible Utilization

**DOI:** 10.3390/antiox14060650

**Published:** 2025-05-28

**Authors:** Ramesh Kumar Saini, Mohammad Imtiyaj Khan, Vikas Kumar, Xiaomin Shang, Ji-Ho Lee, Eun-Young Ko

**Affiliations:** 1 School of Health Sciences and Technology, UPES, Dehradun 248007, India; rameshkumar.saini@ddn.upes.ac.in; 2 Biochemistry and Molecular Biology Lab, Department of Biotechnology, Gauhati University, Guwahati 781014, India; imtiyaj@gauhati.ac.in; 3 Department of Food Science and Technology, Punjab Agricultural University, Ludhiana 141004, India; vikaschopra@pau.edu; 4 Jilin Provincial Key Laboratory of Nutrition and Functional Food, Jilin University, Changchun 130062, China; xmshang@jlu.edu.cn; 5 School of Natural Resources and Environmental Science, Department of Biological Environment, Kangwon National University, Chuncheon 24341, Republic of Korea; micai@kangwon.ac.kr; 6 School of Animal & Food Sciences and Marketing, Konkuk University, Seoul 05029, Republic of Korea

**Keywords:** food waste, valorization, carotenoids, polyphenols, microencapsulation, active and intelligent packaging, circular economy

## Abstract

Domestic food waste and agro-industrial by-products (AIBPs) occurring throughout the food chain, including production, processing, and storage, have become a global sustainability concern. Interestingly, this waste and these by-products contain a significant amount of commercially vital bioactive compounds, including polyphenols and carotenoids. Remarkably, discarded by-products such as fruit and vegetable peels contain more bioactive compounds than edible pulp. Thus, valorizing this waste and these by-products for commercially vital bioactive products can solve their disposal problems and help alleviate climate change crises. Additionally, it can generate surplus revenue, significantly improving food production and processing economics. Interestingly, several bioactive extracts derived from citrus peel, carrot pomace, olive leaf, and grape seed are commercially available, highlighting the importance of agro-food waste and by-product valorization. Considering this background information, this review aims to provide holistic information on major AIBPs; recovery methods of bioactive compounds focusing on polyphenols, carotenoids, oligosaccharides, and pectin; microencapsulation of isolated bioactive for enhanced physical, chemical, and biological properties; and their commercial application. In addition, green extraction methods are discussed, which have several advantages over conventional extraction. The concept of the circular bio-economy approach, challenges in waste valorization, and future perspective are also discussed.

## 1. Introduction

The Food Waste Index Report 2024 by the United Nations Environment Program revealed that 19% (1.05 billion tons, more than USD 1 trillion) of total global food production is wasted through households (59%), food service (27%), and retail (1.82%) [1,2]. This is equivalent to 132 kg of food waste per capita per year. Sustainable Development Goal (SDG) target 12.3 of the UNEP aims at a 50% reduction in per capita global food waste in the consumer and retail sector and a reduction in food losses in production and post-harvest handling.

Three categories can be used to classify waste utilization: upcycling or valorizing (conversion to higher-value products), recycling (converting to similar or lower-value products), and reuse (using it for the same purpose). Food loss and waste are often conflated but represent distinct concepts. Food loss refers to the reduction or exit of edible food or its components from the post-harvest supply chain, rendering them unavailable for further utilization. In contrast, food waste pertains to the disposal of edible or inedible parts, typically at the consumer or retail level.

In addition to the food losses in households, food service, and retail, the fruit and vegetable processing industries generate substantial agro-industrial by-products (AIBPs), such as kernels, peels, and pomace. Some of this waste is utilized in composting and animal feed, while a significant part of this waste is dumped in controlled or uncontrolled landfills, which is a severe environmental concern, as the decomposition of these wastes releases significant amounts of greenhouse gases and can require a more extended period for complete deterioration [3].

AIBPs can be utilized to isolate economically vital compounds, including polyphenols [4,5], carotenoids [6], dietary fiber, organic acids, proteins and peptides, lipids, polysaccharides, enzymes, vitamins, biofuels, animal feed, and essential oils [7,8]. Among these compounds, bioactive compounds, including polyphenols and carotenoids, are in high demand in the pharmaceutical, food, healthcare, and chemical industries [9]. In addition, these bioactive substances are abundant in most fruit and vegetable waste, especially pomace and peels. According to one estimate, 5.4–9.0 million tons of tomato pomace and 10 million tons of citrus peel are produced globally [10,11]. Similarly, 6–8 million tons of crustacean processing waste are produced worldwide [12], an exceptionally rich source of astaxanthin, a carotenoid with a high economic value.

Interestingly, discarded by-products like peel contain more antioxidant compounds than their edible parts [13,14,15]. For instance, in kiwi fruit, two times higher amounts of phenolic compounds were recorded in peel compared to pulp [15]. In another study, kiwi peels exhibited significantly higher contents of tocopherol and organic acids than edible pulp [16]. Similarly, seeds discarded during the domestic processing of cucurbit fruits, such as cantaloupe melon, also contain higher total phenolics and antioxidant activity than pulp [17]. Moreover, on a weight basis, peel contributes 31% of the total phenolic content assessed in cantaloupe melon [17].

Considering the importance of AIBP valorization, several high-quality reviews have been published [5,9,18,19,20,21,22,23]. Corrado and Sala [18] reviewed the studies on food waste generation at the European and global scales. Shahidi et al. [5] provided detailed information on phenolic-rich fruit and vegetable by-products. Sagar et al. [9] explored the nature of the waste originating from fruits and vegetables. Mahato et al. [19] reviewed the nature, production, and types of bioactive compounds derived from fruits and vegetables. Peydayesh et al. [20] provided a roadmap for the utilization of food protein waste in stainable technologies. More et al. [21] discussed the advantages of modern extraction methods over conventional methods for the extraction of bioactive compounds from fruit/vegetable and processing wastes. Reguengo et al. [22] provided an overview of bioactive-rich major agro-industrial by-products. Vilas-Boas et al. [23] highlighted the toxicity and safety concerns associated with compounds isolated from food waste. This review critically and comprehensively discusses all these advancements in the field of AIBP valorization, including their bioactive composition; methods to isolate the bioactive compounds; encapsulation methods; and their applications in food, nutraceuticals, probiotics, stabilizers in vegetable oil, biodegradable packaging material, and the preparation of active and intelligent films. In addition to the bioactive compounds, oil, oligosaccharides, and pectin recovery are also discussed. Furthermore, the extraction of small amounts of bioactive compounds does not eliminate the bulk mass of food waste by-products. A circular bioeconomy concept is vital to address this issue, which is also discussed in this review.

## 2. Literature Search Methodology

Available electronic databases, especially the Web of Science core collection, were searched for studies dealing with bioactive-rich (especially carotenoids and polyphenols) AIBPs, methods to isolate the bioactive compounds from AIBPs (including green extraction), and applications of extracted bioactive compounds.

The primary search keywords were waste valorization (topic) and bioactive (topic). The other keywords were the following: (1) waste valorization (topic) and carotenoid (topic); (2) waste valorization (topic) and polyphenol (topic); (3) waste valorization (topic) and green extraction (topic); (4) waste valorization (topic) and circular bio-economy (topic); (5) waste valorization (topic) and safety (topic); (6) bioactive (topic) and encapsulation (topic); and (7) food waste (title) and bioactive (topic). The relevant 384 articles downloaded were published mainly between 2018 and 2025. A total of 205 articles are discussed in this review.

## 3. AIBPs Are Rich Sources of Bioactive Compounds

**Figure 1:**
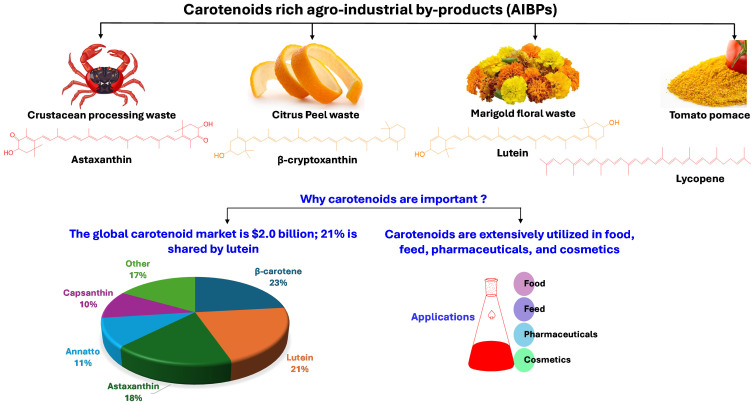
Major carotenoid-rich agro-industrial by-products (AIBPs) and their commercial importance [24].

**Figure 2:**
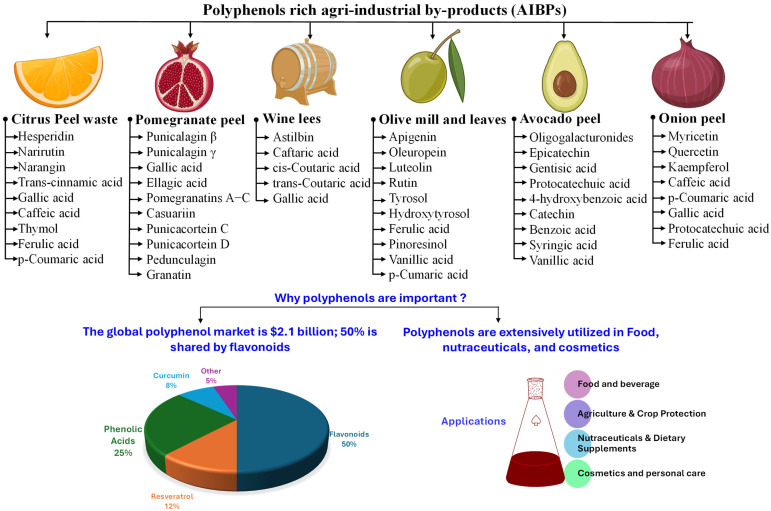
Major polyphenol-rich agro-industrial by-products (AIBPs) and their polyphenol composition.

AIBPs are rich sources of bioactive compounds, particularly carotenoids and polyphenols, which are widely recognized for their antioxidant activity and health-promoting benefits. The key AIBPs containing significant amounts of carotenoids and polyphenols and their economic importance are illustrated in Figure 1 and Figure 2, respectively. Table 1 summarizes the bioactive composition of various AIBPs. The following sections provide a detailed overview of major categories of AIBPs rich in carotenoids and polyphenols.

### 3.1. Fruit and Vegetable Waste

Processing most fruits and vegetables produces by-products, typically 25–30% of the raw materials, in the form of pomace, seed, and peel [21]. These by-products are rich in commercially vital compounds. For instance, olive pomace is a major output of olive oil processing, rich in α-tocopherol (2.63 mg/100 g), oleic acid (75% of total fatty acids), and polyphenols [27]. Hydroxytyrosol (83.6 mg/100 g) and comselogoside represent 53.78% and 25.36% of the total polyphenols in olive pomace, respectively. Moreover, olive pomace skin is a rich source of triterpenic acids, with the content ranging from 140 to 400 mg/g. Maslinic acid accounts for 70% of these total triterpenes [28]. According to estimates, leaves comprise about 10% of the weight of the olives delivered to the mill. These leaves are abundant in triterpenes such as oleuropein, α-amyrin, oleanolic acid, and maslinic acid [29]. Madureira et al. [68] have provided an overview of the extraction of hydroxytyrosol, oleuropein, and tyrosol from olive waste and their applications.

Another important by-product is tomato pomace. When tomatoes are processed in industries, a substantial quantity of tomato pomace (5–30% of the final product) is created as food waste or by-products [6,32], which are primarily used as animal feed or dumped in landfills. The tomato pomace is nearly 35–40% seed and 57–65% skin [33]. Tomato pomace contains a substantial amount of fiber, tocopherols, polyphenols (mainly ellagic and chlorogenic acids, rutin, and myricetin), terpenes, minerals, and sterols, and the skin in particular contains high amounts of lycopene (447–510 µg/g dry weight (DW)) [10,32,33]. In addition, lycopene is the major carotenoid found predominately in the tomato skin, which is discarded during tomato processing. Due to the presence of a significant amount of these bioactive compounds, especially lycopene, tomato pomace exhibits high antioxidant activities. The Trolox equivalent (eq.) antioxidant capacity assay-measured antioxidant activity showed 224.81 µmol trolox eq./100 g DW of tomato pomace [33].

Wine making also generates large amounts of by-products that have an ecological impact. However, these by-products contain a significant amount of polyphenolic compounds with high economic and nutritional significance [69]. Grape skin, seeds, and pomace obtained from wine, juice, and boiled juice processing may constitute 13.5% to 20% of the total volume of grapes and are rich in anthocyanins, tocopherols, minerals, and other bioactives [45]. The second-largest waste from oenological activities is wine lees. Lees are yeast-rich sludges that contain a substantial amount of polyphenols, including hydroxycinnamic acids, flavonols, and anthocyanidins, which are produced during the vinification process [47].

### 3.2. Citrus Processing Waste

Citrus fruits (genus *Citrus* L., family Rutaceae) are among the most widely cultivated crops worldwide. According to an estimate, 158 MT of citrus fruits are produced globally [70]. The juice processing industries utilize nearly 40% of this produce [71], generating 50–60% of waste, comprising seeds, peel, and pulp, which are collectively called pomace [72,73]. This citrus pomace contains a considerable amount of commercially vital phytochemicals such as pectin, organic acid, dietary fiber, limonoids, polyphenols, carotenoids, and vitamins [19,72,74,75]. It has been assessed that 51.8 million tons of oranges are produced globally per year, with a substantial fraction destined for industrialization [76]. After processing, 50–60% of these oranges, which are composed of mainly segment membrane, peel, and seeds, end up as waste [76].

Peel forms the major fraction of citrus pomace, which is rich in carotenoids [77,78,79], essential oils (limonoids) [80], flavonoids [81,82,83], pectin [84,85], and several other commercially vital bioactive compounds with potent antioxidant [81] and health-beneficial properties [73,86,87,88]. Among the flavonoids, naringin, hesperidin, neohesperidin, and rutin are present in significant amounts in citrus fruit peel [75,83,88], especially mandarin peels, which exhibit high antioxidant activities [83]. Hesperidin (85–350 mg/100 g DW), naringin (26–244 mg/100 g DW), and phloretin (14–40 mg/100 g DW) are the major flavonoids in yuzu (*Citrus junos* Sieb ex Tanaka) peel [4]. In addition, citrus fruits contain unique polymethoxylated flavones (e.g., tangeritin, 5-demethyl nobiletin, and nobiletin), which are well known for their health-beneficial properties [89]. Furthermore, citrus peel is abundant in essential oils containing aliphatic aldehydes and oxygen-containing mono- and sesquiterpenes [90]. In the Montenegrin mandarin fruit peel essential oil, γ-terpinene and D-limonene were the significant fractions, with minor occurrence of β-linalool and citronellol [91].

Citrus peel is composed of flavedo (outer layer of the peel) and albedo (white, spongy material in citrus fruits located between the juicy pulp and the peel), which contain significantly higher amounts of polyphenols and other bioactive compared to juicy pulp [14,78,92]. Apocarotenoids and carotenoids confer the orange/red color to the pulp and peel of citrus fruits [93]. In particular, β-cryptoxanthin is a major citrus carotenoid with provitamin A activity [94,95]. Like the polyphenols, the citrus fruit peel flavedo contains more carotenoids than juice sacks [92]. High levels of polyphenols in the peel have a phytoprotective effect, protecting the fruits from phytopathogens and other stressors like wounds, intense sunlight, and cold temperatures [96]. In our study, the peels of six citrus species showed substantially higher total phenolic and flavonoid content and antioxidant potential than edible pulp [13].

Similar to peel, citrus seeds are rich in health-beneficial phytochemicals, including ascorbic acid [78], proteins [87], dietary fibers, flavonoids, limonoids, tocopherols, fatty acids, and phytosterols [78,97]. A comparative study among peel, seeds, and pulp of fruits of various varieties of mandarins, including kumquat (*C. japonica*), clementine (*C. clementina*), and Phlegraean mandarin (*C. reticulata*), revealed that Phlegraean mandarin seeds are richer in bioactive compounds, including antioxidant activity, total polyphenols, ascorbic acid [78].

### 3.3. Crustaceans and Fish Processing Waste

Industrial and domestic processing of shrimp to separate edible flesh generates 38.1–45.4% head and carapace residues (waste), rich in astaxanthin, tocopherols, and omega-3 (n3) long-chain (LC) polyunsaturated fatty acids (PUFAs), including docosahexaenoic acid (DHA; C22:6) and eicosapentaenoic acid (EPA; C20:5) [6,25]. Interestingly, in our study, an equal amount of LC-n3-PUFAs and a significantly higher amount of α-tocopherol (32.0–35.3 µg/g FW) was recorded in shrimp processing waste than edible flesh (16.4–21.7 µg/g FW of α-tocopherol) [25]. In another study, oil obtained from sardine (Sardina pilchardus) waste including viscera, spines, and heads generated in a canning industry was found to contain 17.2% (*w*/*w*) PUFAs [26].

### 3.4. Other Waste

Lignocellulosic biomass is the most abundantly available biomass. For instance, the cocoa pod husk is the main by-product (70–75% weight of whole fruit) of the cocoa harvest and is rich in minerals (mainly potassium, 2.8–3.8% *w*/*w*), fiber (including cellulose, hemicellulose, pectin, and lignin), and polyphenols [48]. Similarly, cocoa shells, the outer portions of beans, are a waste produced from the roasting process during chocolate production. This by-product is rich in fibers and phenolic compounds, especially catechin and hydroxybenzoic acid [50].

Potato processing generates 6–10% peel, which is discarded as waste. However, it contains a significant amount of polyphenolic compounds and dietary fiber [30]. Similarly, pomegranate peel, a by-product obtained after juice extraction, comprises 30–40% of the pomegranate fruit. It is a rich source of phenolic acids (e.g., caffeic, chlorogenic, syringic, ferulic, and gallic acid), flavonoids (e.g., anthocyanins, catechin, quercetin, and epicatechin), and tannins (e.g., ellagic acid and ellagitannins derivatives) [41].

Edible mushrooms and their by-products (spent mycelium substrate) have a high content of carbohydrates, proteins, dietary fiber, polyphenols, fatty acids, vitamins, and minerals [51]. Moreover, mushrooms are well known for their potent antimicrobial activities, linked to the presence of polyphenols, phytosterols, carotenoids, and ascorbic acid [51].

## 4. Recovery of Bioactive Compounds from AIBPs

### 4.1. Processing of AIBPs

AIBPs contain a significant amount of moisture (e.g., tomato pomace contains 55.58% moisture [33]), which provides a favorable environment for the growth of microbes. Thus, dehydration of agrifood by-products is performed to increase their shelf-life and reuse potential. Drying reduces the activity of enzymes and microbes significantly, increases shelf life, facilitates the handling and distribution of a by-product, and yields a number of functional ingredients with added value that can be utilized in the food industry [98]. However, drying time and temperature can affect the stability of phytochemicals. Additionally, drying can substantially increase the processing cost of the product. Lyophilization (freeze-drying) methods are well known for retaining the maximum phytochemicals. In addition, they prevent browning and off-flavor development during the dehydration. However, the running and initial cost of the instrumentation is very high compared to other drying methods, such as hot air drying.

Hot air drying is the most common method of dehydration used in food industries because of its low processing and investment costs. For instance, when used in the processing of vegetable wastes, it offers integral valorization of the biowastes [99]. Cecchi et al. [98] compared the polysaccharides and phenols profile of olive pomace (destoned and partially dehydrated) and pomegranate peel dehydrated using a lab-scale oven (50–110 °C), industrial drying (150 °C), and freeze-drying. The results showed that industrial drying at high temperatures for a short time provided a dehydrated product with the same properties as the freeze-dried samples, with no browning or off-flavor development, compared to oven drying at low temperatures for longer.

### 4.2. Recovery of Polyphenols

**Figure 3:**
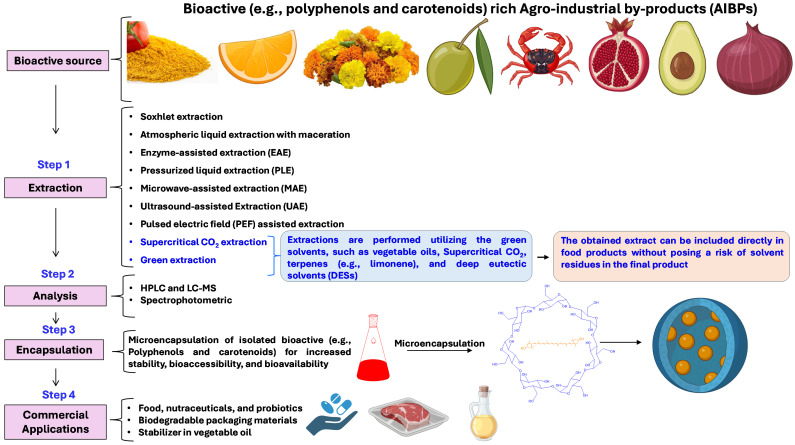
Methods used for the recovery of bioactive compounds from AIBPs and their downstream processing and applications.

Various conventional (e.g., maceration, Soxhlet) and modern extraction techniques, assisted by pulsed electric field, microwave, ultrasound, high pressure, and enzyme, have been investigated to recover the polyphenols from AIBPs in an effective manner (Figure 3, Table 2). In addition, subcritical water extraction (SWE) and supercritical carbon dioxide (CO_2_) extraction (SCE) are emerging as green methods for the recovery of bioactive compounds. Moreover, ultrasound-assisted extraction (UAE), microwave-assisted extraction (MAE), and SCE have been established commercially in a majority of countries across the world because of their efficiency, wide applicability to diverse classes of economically vital compounds, and environmentally friendly nature, which aligns with sustainable extraction practices [100].

In addition to the extraction methods, the extraction effectiveness of bioactive compounds largely depends on the sample matrix, solvent nature, solvent-to-sample ratio, extraction time, and extraction temperature. Aqueous ethanol and acetone-based solvents are most widely utilized in extracting polyphenols from plant samples [4,101,102]. Gullón et al. [101] tested 50% ethanol, 50% acetone, 20% ethanol, 1% NaOH, and water to extract the phenolic compounds from olive tree pruning and olive mill leaf samples with the fixed parameters of solid/liquid ratio of 1:6 (*w*/*v*) and extraction temperature of 55 °C for 90 min. In the results, 50% ethanol and 50% acetone provided the highest yield of phenolic compounds, while water was the least effective.

Soxhlet extraction with ethanol provided the highest extraction yield from Brazilian and Mexican avocado seeds and peels, compared to hexane and ethyl acetate as solvents [103]. In this study, SCE provided a higher yield of total phenolic contents (TPC) from the avocado seeds, while higher values of antioxidant activity were obtained using Soxhlet extraction. Extraction utilizing 50% ethanol (*v*/*v*), a liquid-to-solid ratio of 20 mL/g, and 180 min extraction time provided the highest yield of phenolic compounds and antioxidant potential from dehydrated yerba mate (*Ilex paraguariensis* A.St.-Hil.) leaves [102]. Using response surface methodology (RSM), the optimal extraction settings for the isolation of antioxidants and the major flavonoids, including naringin, hesperidin, and phloretin, from the peels of yuzu fruits were optimized at 65.5% ethanol in water, extraction temperature of 43.9 °C, extraction time of 119.7 min, and solvent-to-dry sample ratio of 37.2 mL/g [4].

**Table 2:** Methods investigated to extract bioactive compounds from agro-industrial by-products (AIBPs).

**AIBPs**	**Extraction Method**	**Bioactive Compounds**	**References**
*Sicana odorifera* fruit epicarp	Heat-assisted extraction for 62 min, 90 °C, 27% ethanol, and UAE (23 min, 500 W, 40% ethanol)	Anthocyanin, 200–281 mg total anthocyanin content/g extract	[104]
Cocoa shells	UAE using 30:49:21 (*v*/*v*) hexane/ethanol/water ratio with 15 min extraction time at 150 W, 19.9 kHz, and 40 °C	Gluconic acid, citric acid, protocatechuic acid, procyanidin, catechin, epicatechin and hydroxybenzoic acid, linoleic acid, and oleic acid	[50]
Sardine (*Sardina pilchardus*)	Oil extraction with SCE with CO_2_ at 25 MPa and 40 °C to obtain the oil; supercritical water extraction at 90–250 °C to obtain fish protein hydrolysate	Oil rich in EPA (4.4%) and DHA (12.8%); fish protein hydrolysate rich in amino acids (404–818 mg/g)	[23]
Wine lees (oenological waste)	Water extraction at 40 °C for 30 min stirring, with the lees/water ratio of 1:10 (*v*/*v*), followed by ultrafiltration using 30 kDa polyacrylonitrile (PAN) membrane	Astilbin, caftaric acid, cis-coutaric acid, trans-coutaric acid, and gallic acid	[47]
Orange, lemon, and clementine peel	Solid-liquid extraction utilizing a ethanol/water ratio of 2:3 (*v*/*v*) for orange and lemon and 1:4 for clementine at 90 °C for 15 min	Hesperidin (280–673 mg/g), naringin, trans-ferulic and p-coumaric acid	[75]
Tomato processing waste (peel and seeds)	Ultrasonic and enzymatic pretreatment with ultrasonic time of 60 min, pectinase concentration of 0.8 g/100 g DW, cellulase concentration of 2.5 g/100 g DW, and pH of 5.3 utilizing 2:1:1 (*v*/*v*) hexane/acetone/methanol	Lycopene (94.3 mg/kg DW)	[31]
Olive tree pruning and olive mill leaves	Extraction utilizing 50% ethanol or 50% acetone with a solid/liquid ratio of 1:6 (*w*/*v*) and extraction temperature of 55 °C for 90 min	Rutin, luteolin, and its mono- and di-glucoside derivatives, and derivatives chrysoeriol (glucoside) and apigenin (rutinoside)	[101]
Tea waste	Steam explosion pretreatment to destroy and restructure the porous network of waste resulted in the increased solubility and extractability of metabolites	Steam explosion enhanced polyphenols, caffeine, saponin, water-soluble sugars recovery, and antioxidant activity by 15.5, 14.1, 28.8, 74.8, and 20%, respectively	[105]
Olive leaf	Polyphenol extraction by temperature-swing adsorption followed by solvent-resistant nanofiltration utilizing polybenzimidazole-based membranes	95–99% pure polyphenols, namely oleuropein, luteolin, and pinoresinol	[106]
Red pitaya (*Hylocereus costaricensis*) peel	UAE at 487 W power for 38 min	Betacyanin (36 mg/g dw), phyllocactin, isophyllocactin, isobetanin, betanin, oxalic acid malic acids, γ-tocopherol (11.8 mg/100 g dw), α-tocopherol (3.10 mg/100 g dw), δ-tocopherol and β-tocopherol	[52]
Olive oil mill wastes	Extraction utilizing ethyl acetate	Tyrosol (12.9 g/L extract), hydroxytyrosol (1.22 g/L extract), oleuropein (2.1 g/L extract), caffeic acid, verbascoside, luteolin, vanillic acid, p-cumaric acid, ferulic acid, and apigenin,	[107]
Acerola (*Malpighia emarginata* DC.) pomace	SWE at a water flow rate of 4 mL/min, 10 MPa, and temperature of 130 °C	Kaempferol, isorhamnetin, quercetin, and ascorbic acid	[53]
Pomegranate (*Punica granatum* L.) peel	PLE at 10.34 MPa pressure, temperature of 200 °C and 77% ethanol	Punicalagin β, Punicalagin γ, Gallic acid, and Ellagic acid	[108]
Avocado peel	Hydrothermal treatment at 150 °C for the highest recovery of total oligosaccharides and 170 C° for the highest recovery of TFC, TPC, and antioxidant activities	Oligogalacturonides, gentisic acid/protocatechuic acid, benzoic acid, 4-hydroxybenzoic acid, syringic acid, vanillic acid, procyanidin dimer, catechin, and epicatechin	[109]
Pomegranate seed	Protease treatment at a concentration of 50 U/g seeds for 14 h, at pH of 7.2 and 45 °C	Oil, proteins, and dietary fiber	[110]
Mango peel	SCE at 25.0 MPa, 15% (*w*/*w*) ethanol at 60 °C	β-carotene (1.9 mg/g DW)	[54]
Pomace press-cake	Aqueous enzyme-assisted extraction utilizing 1.2 units of alkaline protease/100 g press-cake, pH 9, 60 °C, 2 h	Polyphenols, fatty acids, tocols (α-tocopherol, γ-tocopherol), and phytosterol (β-sitosterol)	[111]
Onion (*Allium cepa* L.) peel	Extraction utilizing DES solvent choline chloride/urea (molar ratio of 1:2), extraction time of 120 min at 60 °C, and solvent-to-sample ratio 50:1	Quercetin (6.19 mg/g), myricetin (0.16 mg/g), and kaempferol (0.35 mg/g)	[56]
Orange peel	Extraction utilizing the DES solvent choline chloride/ethylene glycol (1:4) at a temperature of 60 °C, solid-to-liquid ratio of 1:10, and 100 min extraction time	TPC of 3.61 mg GAE/g of orange peel. gallic acid, p-coumaric acid, caffeic acid, thymol, trans-cinnamic acid, and ferulic acid	[112]
Kiwifruit pomace	SWE under 200 °C, extraction time of 90 min, and the extraction pressure of 5 MPs	(+)-catechin, protocatechuic acid, p-coumaric acid, caffeic acid, and chlorogenic acid,	[58]
Jackfruit (*Artocarpus* *heterophyllus* Lam.) peel	Ultrasonic microwave-assisted extraction using 63% ethanol, solvent-to-solid ratio of 34:1, 160 W microwave power, and 20 min irradiation time	TPC (8.14 mg GAE/g DW), catechin, gallic acid, and chlorogenic acid	[59]
Papaya seeds	SWE at 150 °C for 5 min	3,4 Dihydroxybenzoic acid, 4-hydroxymethylbenzoic acid, ferulic acid, chlorogenic acid, gallic acid, methoxyphenylacetic acid, salicylic acid, vanillic acid, myricetin, and resveratrol with the TPC of 417 µg/g of extract.	[61]

Abbreviations: DES, deep eutectic solvent; DHA, docosahexaenoic acid; DW, dry weight; EPA, eicosapentaenoic acid; GAE, gallic acid equivalent; PLE, pressurized liquid extraction; SWE, subcritical water extraction; SCE, supercritical carbon dioxide (CO_2_) extraction; SWE, subcritical water extraction; TFC, total flavonoid contents; TPC, total phenolic contents; and UAE, ultrasound-assisted extraction.

In a comparative study, 50% ethanol (*v*/*v*) was more efficient than 20 and 80% ethanol for extracting polyphenols from olive pomace and wine residues [113]. The extraction performance was not found to be dependent on the presence of hydrochloric acid (0–0.5%). Moreover, in this study, the highest extraction efficiency from olive pomace residues was delivered by MAE (90 °C, 5 min), and for wine residues by PLE (100 °C, 5 min, one cycle). However, the results for the UAE for 30 min were also suitable. UAE is recommended for future scaling-up evaluations considering extraction performance, initial investment, and running costs. In another study, 55% ethanol was the most efficient, compared to 75% and 100% ethanol, for extracting polyphenols from lime (*Citrus aurantiifolia*) peel [114]. Moreover, in this study, UAE (38% amplitude for 4 min) was more effective for extracting the TPC (54 mg gallic acid eq. (GAE)/g), with significantly high antioxidant activity and time saving of 33% compared with MAE (140 W for 45 s with eight repeats). A study by Vu et al. [115] on MAE of polyphenols from banana peel revealed that 50.55 mg polyphenols (gallic acid equivalent) could be recovered from 1 g dried peel utilizing the RSM-optimized condition of pH of 1, sample-to-water ratio of 2:100 g/mL, microwave power of 960 W, and 6 min irradiation time. Based on the various studies reviewed above in this section, aqueous ethanol seems to be the most effective solvent for phenolic compound extraction, irrespective of the matrix, phenolic compound composition, and other factors.

Narirutin and hesperidin are the key flavonoids found in *C. unshiu* peel [116,117]. Hwang et al. [74] used the pulsed electric field (PEF) and SWE methods to optimize the extraction of narirutin and hesperidin from the peel of *C. unshiu*. This study obtained the maximum 46.96 mg/g DW of hesperidin from SWE treatment at 150 °C for 15 min combined with PEF for 2 min. The highest yield of narirutin of 8.76 mg/g DW was obtained from SWE at 190 °C for 5 min combined with PEF treatment for 2 min. In another study, for the semi-continuous SWE of narirutin and hesperidin, a recovery rate of 90–94%, an ideal flow rate of 2.25 mL/min, and ideal extraction temperatures of 154.6 °C (narirutin) and 164.4 °C (hesperidin) were predicted using RSM [117]. Similarly, SWE of polyphenols from onion skin in a semicontinuous extractor (105–180 °C, 2.5 mL/min, and 5 MPa) showed the highest extraction yield at 145 °C within a short duration of less than 30 min. In the extract, quercetin (15.4 mg/g DW of skin waste) and quercetin-4′-glucoside (8 mg/g DW) corresponded to 90% of the total flavonoids identified. Interestingly, in this study, precipitation of high-temperature-extracted flavonoids observed during the extract cooling was successfully eliminated by adding ethanol to increase the polarity of the water to be similar to water at 180 °C [118].

Ultrasound-assisted aqueous two-phase extraction provided the highest yield of flavonoids from jujube peels with the RSM-optimized extraction solvent of 35% K_2_HPO_4_ and 20% ethanol (*w*/*w*), solid–liquid ratio of 1:30 g/mL (*w*/*v*), extraction time of 50 min, and ultrasonic power of 200 W [119]. Rutin, kaempferol-3-O-rutinosid, and quercetin 3-β-D-glucoside were identified as the main flavonoids in the extract. In addition, ultrasound promoted the mass transfer, and hydrogen bonding, salting out effect, and van der Waals force were identified as the prominent mechanisms behind the efficient extraction of jujube peel flavonoids. In another study, Nunes et al. [27] compared the extraction efficiency of olive pomace polyphenols utilizing UAE using different input electric power and extraction times of 90 W/10 min, 160 W/10 min, and 160 W/5 min, with the conventional solid–liquid extraction method (60 min). The results showed the highest extraction at 160 W/5 min, followed by 160 W/10 min, 90 W/5 min, and conventional extraction. The RSM-optimized UAE of betacyanins at 487 W for 38 min provided the maximum betacyanin content of 36 mg/g DW from red pitaya (*Hylocereus costaricensis*) peel [52]. The betacaynins were phyllocactin, isophyllocactin, isobetanin, and betanin.

Using high temperature and a high percentage of water improves the diffusion rates (elevated temperature furthers the matrix–analyte interactions) and solubility of phenolic compounds, resulting in higher extraction efficiency [120]. On the other hand, elevated pressure helps in keeping the solvent below its boiling point in conditions of high temperature. From fruit and vegetable residues obtained after juice extraction, the highest extraction yield was obtained at 200 °C with 1:1 ethanol/water (*v*/*v*) [120]. In a comparative study, hot methanol extraction provided a higher yield of hesperidin from wet albedo (white spongy interior of orange *Citrus sinensis* L. osbeck peel) compared to dehydrated albedo [121].

Ruan et al. [40] isolated ten phenolic compounds utilizing ethanol/water (5:5, *v*/*v*) with three successive extractions at sample-to-solvent ratios of 1:10, 1:5, and 1:50 at 50 °C. The obtained extract was fractionated using nuclear magnetic resonance and preparative/semipreparative high-performance liquid chromatography techniques. The compounds were identified as pomegranatins A–C, D-glucopyranose, gemin D, casuariin, punicacortein D, punicacortein C, pedunculagin, and granatin, from pomegranate (*Punica granatum* L.) peel. These compounds exhibited antimicrobial, DPPH radical scavenging, and antitumor activities toward the HeLa cell line.

Polyphenols in plant biomass are bound to cell wall polysaccharides by hydrogen bonds and hydrophobic interactions. Therefore, cellulase, pectinase, and tannase degrade this complex matrix and release the associated polyphenols [122]. Specifically, in syrah grape pomace, it was observed that tannase degrades hydrolyzable tannin, liberating primarily gallic acid. At the same time, cellulase hydrolyzes cellulosic fibers and favors the liberation of malvidin-3-O-glucoside and p-coumaric acid [122]. Overall, in this study, optimized aqueous extraction assisted by enzyme extraction using 198 U of tannase/g of grape pomace and 188 U of cellulase/g of grape pomace at pH 5.0 and at 45 °C improved the yield of TPC by up to 66% [122]. In another study of enzyme-assisted extraction of non-extractable polyphenols from sweet cherry (*Prunus avium* L.) pomace, the Promod enzyme (from *Humicola* sp.) followed by Depol (from *Bacillus licheniformis*) enzyme was more efficient than pectinase (from *Aspergillus* sp.) [123].

In a comparative study, PLE using an aqueous solution assisted with cellulase enzymes provided the highest recovery of caffeine (46 g/100 g extract), catechin (51 g/100 g extract), TPC, and antioxidant potential from crude guarana (*Paullinia cupana*) seeds, compared to PLE alone using 50% ethanol, SCE, and SCE associated with enzymes [124]. The low solubility of the targeted compounds (catechins and methylxanthines) in CO_2_, attributed to differences in polarity, probably caused the low extraction yield in SCE.

Steam explosion pretreatment was shown as a potential strategy to destroy and restructure the porous network of tea waste, resulting in increased solubility and extractability of metabolites [105]. Steam explosion pretreatment was potentially effective in destroying tea waste’s major cell wall components by removing hemicellulose and redistribution of lignin. This process enhanced the recovery of polyphenols, caffeine, saponin, and water-soluble sugars and antioxidant activity by 15.5, 14.1, 28.8, 74.8, and 20%, respectively.

In recent years, integrating extraction techniques has provided advantages in terms of enhanced mass transfer rate with reduced extraction time, even at low temperatures [63]. For instance, phenolic compounds have been extracted from passion fruit rind using ultrasound-assisted pressurized liquid extraction, which offers advantages over PLE and UAE [63]. With this integrated method at an extraction temperature of 60 °C, a pressure of 10 MPa, a solvent flow rate of 10 g/min, and ultrasonic intensity of 360 W/cm^2^, 100% of TPCs were recovered in 68.54 min of extraction time. Moreover, these extraction conditions provided the lowest manufacturing costs of USD 245/ g TPC.

A temperature-swing adsorption system was utilized to extract major polyphenols, namely oleuropein, luteolin, and pinoresinol, from olive leaf waste [106]. This process was followed by solvent-resistant nanofiltration (“in-line” concentration) utilizing polybenzimidazole-based membranes to recover highly pure (95–99%) polyphenols, as well as >97% of the solvent. Most importantly, this optimized process resulted in a 49% and 61% reduction of the carbon and ecological footprints, respectively. Thus, such a system can be adapted to isolate high-purity bioactive compounds sustainably.

Mir-Cerdà et al. [47] investigated ultrafiltration (UF) for clarification and fractionation to separate the polyphenols from wine lees (oenological waste). This study recovered flavonoids, phenolic acids, and related compounds from this oenological waste utilizing water as the solvent at 40 °C for 30 min stirring, with a lees/water ratio of 1:10 (*w*/*w*). In this study, the obtained extract was subjected to ultrafiltration (UF) utilizing polyacrylonitrile (PAN) and polyethersulfone (PES) membranes of 30 and 5 kDa cut-off, respectively, to eliminate microparticles. The PAN 30 kDa membrane more efficiently eliminated the macromolecules and microparticle contaminants without losing polyphenols.

From mango processing waste containing a mixture of puree, peels, and seeds, the maximum concentrations of mangiferin (354.4 mg/kg DW) and hyperoside (quercetin 3-O-galactoside, 258.7 mg/kg) were obtained utilizing an RSM-optimized sample/solvent ratio of 29.33% and 28.17%, ethanol/water concentration of 67.73% and 70.11%, and time of 4.47 min and 5.00 min, respectively [125].

Studies have indicated that the majority of extracted and reported polyphenols consist of free and non-bound fractions and not non-extractable polyphenols (NEPs) [126,127]. This is mostly because residues after initial non-hydrolytic extraction are discarded rather than being subjected to further hydrolysis for NEP recovery. Without the hydrolytic pretreatments, NEPs, such as proanthocyanidins and hydrolyzable tannins, remain sterically hindered in a network of cellular structures, such as cell wall components and macromolecules including proteins and polysaccharides. Grains, grape, and orange by-products are rich sources of non-extractable polyphenols [127]. Hydrolytic acid, alkaline, or enzyme pretreatments are required to extract these non-extractable polyphenols [127].

### 4.3. Recovery of Carotenoids

AIBPs are a rich source of commercially vital carotenoids, especially fruit pomace (e.g., tomato, carrot, mango, papaya) and crustacean processing waste [128]. In addition to the AIBPs, undersized fruits containing a substantial amount of carotenoids are discarded. For instance, a significant amount of undersized and damaged bell peppers, rich in carotenoids and polyphenolic compounds, are discarded. Total carotenoid contents of 82.8–103.6 µg/g DW were recorded in the edible portion of yellow bell pepper (*Capsicum annuum* var. Kioto yellow pepper) waste [64]. Violaxanthin (32.17–50.87 µg/g DW), lutein (12.00–26.00 µg/g DW), β-carotene (11.73–12.65 µg/g DW), and zeaxanthin (9.00–15.40 µg/g DW) were the major carotenoids. In addition, 2.77–3.2 mg/g DW of phenolic compounds were recorded in this pepper waste.

A large number of methods are available for the extraction of carotenoids from food processing waste (Figure 3, Table 2). PEF extraction has shown promising results in maximizing carotenoid yield with low energy utilization [129]. PEF treatment of tomato residue at 1.0 kV/cm for 500 pulses with total energy inputs of 28.5 kJ/kg improved the lycopene extraction yield from 9.84 to 14.31 mg/100 g residues, compared to the control, with a significant increase in the yield of total carotenoids (56.4% increase), TPC, proteins, and antioxidant capacity [129]. PEF causes rapid cellular disintegration, which leads to increased extractability of intracellular compounds within a short duration, thus minimizing the energy and solvent requirements. In another study, PEF treatment (5 kV/cm, 5 kJ/kg) of industrial tomato peel residues significantly enhanced the lycopene yields (12–18%; predominantly all-*E*-lycopene) and lycopene extraction rate (27–37%), and the antioxidant potential of extract using acetone and ethyl lactate. Tomato peel micrographs revealed that PEF caused plant cells to shrink in size and separate from one another, most likely as a result of hole creation and intracellular substance leakage.

Among the tomato peel extracts of ten varieties screened for their carotenoid and phenolic contents, the highest contents of total carotenoids, 5.31 mg/100 g DW, were recorded in a local variety, Taranesti roz, while the highest TPCs of 155 mg/100 g DW were recorded in a commercial hybrid, Mirsini [130]. In this study, in Taranesti roz, lycopene was the major carotenoid (3.70 mg/100 g DW), followed by β-carotene (0.53 mg/100 g DW), and lutein (1.09 mg/100 g DW). Among polyphenols, 3,4-di-O-caeoylquinic acid was the most dominant (15.7 mg/100 g DW), followed by quercetin-3-rutinoside (11.4 mg/100 g DW), and naringenin chalcone (9.4 mg/100 g DW) in Mirsini.

Rahimpour and Dinani [31] optimized the four independent variables, including ultrasonic time (60 min), pectinase concentration (0.8 g/100 g DW), cellulase concentration (2.5 g/100 g DW), and pH (5.3) to obtain the highest extraction yield of lycopene (94.3 mg/kg DW) from tomato processing waste (peel and seeds) utilizing the ultrasonic pretreatment–enzymatic process with 2/1/1 (*v*/*v*/*v*) hexane/acetone/methanol.

The ethanol concentration in the CO_2_ plays a significant role in the SCE extraction of carotenoids [54]. SCE at 15% *w*/*w* ethanol, 60 °C, 25.0 MPa provided the highest yields of β-carotene (1.9 mg/g DW) and total carotenoids from mango peel [54]. SCE under optimized conditions of 15 g/min CO_2_, 15.5% (*v*/*v*) ethanol as co-solvent, 35 MPa, 59 °C, and 30 min of extraction time provided >90% of total carotenoid recovery from diverse vegetable matrices, including apricot, sweet potato, peach, tomato, and pumpkin, as well as flesh and wastes of red, yellow, and green peppers [131]. In this study, the authors observed that the presence of >11% lignin in the sample matrix (e.g., 13.3% in pumpkin peel) hinders the extraction efficiency of SCE. Lignin, which fills up the spaces between hemicellulose, cellulose, and pectin in the plant cell wall, creates a robust physical barrier for CO_2_ penetration in SCE.

Despite the large number of methods available for the extraction of carotenoids from food processing waste, to date, there is not an extraction method that is economically viable compared to the synthetic production of carotenoids.

### 4.4. Novel Extraction Methods

Bioactive compounds are commonly extracted utilizing a mixture of organic solvents. However, in recent years, green solvents, such as vegetable oils, supercritical CO_2_, terpenes (e.g., limonene), and deep eutectic solvents (DESs) have been utilized for the extraction of carotenoids, tocopherols, and polyphenolic compounds from fruit and vegetable by-products [132,133]. The name “green extraction” is due to using renewable natural products, less energy consumption, less time in the extraction process, and minimum use of hazardous substances [134]. Moreover, green extraction processes have the potential to be used on an industrial scale [21]. Most importantly, green solvents provide potential advantages in bioactive extraction by minimizing the requirements of the downstream process of solvent elimination, as the obtained extract can be directly utilized without posing a risk of solvent residues in the final product.

The quaternary ammonium salt, metal salt hydrate, metal salt, and hydrogen bond donor are the common types of DESs utilized to extract value-added components from agro-food waste [135]. DESs are composed of natural compounds, including amino acids, organic acids, choline derivatives, and sugars. They are usually termed natural deep eutectic solvents (NADESs) [135]. Fernández et al. [136] compared the efficiency of NADESs such as citric acid/glucose, lactic acid/glucose, and fructose/citric acid with conventional solvents (methanol and water) to extract the polyphenols from olive, onion, pear, and tomato industrial by-products. In the results, NADES based on lactic acid/glucose with 15% water showed the highest extricability of both weak polar and polar polyphenolic compounds compared to conventional solvents (e.g., water and methanol). Moreover, in the stability test, phenolic compounds belonging to various classes that are unstable in aqueous solutions (degrade up to 90% in two months) showed improved stability in NADES—suggesting their possible utilization as vehicles of bioactive compounds in food products.

Choline chloride–malic acid showed the highest extraction yield of polyphenolic compounds from *Carya cathayensis* Sarg. peels [137]. In addition, in this study, extracts obtained from these NADESs showed antioxidant, α-amylase, α-glucosidase, and inhibition activities. A molecular dynamic simulation analysis study showed that more hydrogen bonds between choline chloride–malic acid and extract, larger solvent-accessible surface area, lower intermolecular interaction energy, and longer lifetime of the hydrogen bonds contributed to the higher extraction of polyphenolic compounds.

Among the several DES solvents tested for the UAE extraction of polyphenolic compounds from spent coffee grounds, the highest yield was obtained utilizing the 67.5% HC-6 (1,6-hexanediol/choline chloride in a molar ratio of 7:1) at 60 °C. [138]. Moreover, these DESs provided a higher yield of polyphenolic compounds than water or aqueous organic solvents.

DES outperforms aqueous ethanol by extracting polyphenolic compounds from orange peel [112]. Structural analysis of orange peel biomass suggested DES as an efficient solvent for cell wall dissolution, allowing efficient mass transfer of target metabolites [112]. Optimum conditions of 10% DES (choline chloride/ethylene glycol, 1:4, in water), at 1:10 solid/liquid ratio, extraction time of 100 min, and temperature of 333.15 K provide the highest TPC (3.61 mg GAE/g of orange peel) and the highest antioxidant potential. In contrast, in this study, ethylene glycol provided the highest TPC of 5.84 mg GAE/g of orange peel [112].

Among the various green solvents evaluated as hexane replacements for the recovery of limonene from orange peel, cyclopentyl methyl ether at optimized conditions of extraction temperature of 70 °C, extraction time of 150 min, and solid/liquid ratio to 10 provided the highest yield, compared to hexane, 2-methyl-tetrahydrofuran, ethyl acetate, isopropyl alcohol, ethyl lactate, polyethylene glycol 300, isopropyl acetate, methyl ethyl ketone, and dimethyl carbonate [139]. In this study, structural analysis of orange peel biomass by scanning electron microscopy revealed the efficient structural disruption of biomass, allowing efficient mass transfer of limonene in the extraction medium.

In a study of green oleo-extraction of volatile (e.g., o-cymene, borneol, camphor, eucalyptol, limonene, terpinen-4-ol, and α-pinene) and non-volatile (e.g., carnosic, rosmarinic acid, and carnosol) bioactive compounds from rosemary leaves using vegetable oils and their amphiphilic derivative, refined soybean oil yielded the highest yield, compared to 11 other types of refined vegetable oils [140]. Moreover, in this study, adding soy lecithin (1%, *w*/*w*) significantly enhanced the extraction efficiency of polar and volatile aroma compounds.

Ethanol percentage and extraction pressure are the critical factors influencing the yield of the target compounds in SCE [141]. The SCE extraction at 13.7% ethanol, 29.9 MPa pressure, and 60 °C provided the highest extraction yield of 0.52%, containing 0.213 mmol trolox eq./g extract and 12.97 mg GAE/g extract and from cacao pod husk [141].

### 4.5. Techno-Economic Assessment of Polyphenols and Carotenoids Recovery from AIBPs

The successful valorization of AIBPs primarily depends on the techno-economic potential. Arias et al. [142] performed a techno-economic analysis to evaluate the feasibility of phenolic production scenarios for AIBPs valorization, including orange peels and tomato seeds, with the production capacity of 100 tons per year of the final product in powder form, utilizing a different extraction methodology. The results showed that the valorization of orange peels is more profitable due to the higher concentration of phenolics than tomato seeds. Orange peel vaporization using conventional solvent (ethanol), UAE, MAE, and SWE provided 8.55, 8.50, 26.88, and 31.70 mg/g of polyphenols. Among all these methods, using MAE, the orange peel valorization process provided the minimum selling price of the final product of USD 0.01/g. This lowest cost was due to a shorter extraction time (only 3 min) and a lower temperature, corresponding to a lower energy demand. Moreover, due to the reduced extraction time, the size and capacity of the equipment required for the extraction were also lower, significantly reducing the equipment costs.

In a case study from Argentina, extracting polyphenols and biogas from orange peel waste was economically feasible under the proposed conditions, with a minimum processing scale of 16 tons/h [143]. The production of polyphenolic-rich extract from poplar (*Populus nigra* × *Populus maximowiczii* (L.)) biomass utilizing SCE was found to be profitable with a production cost (break-even price) of EUR 145/kg of extract [144].

Co-extraction of lycopene and pectin from pink guava (*Psidium guajaya*) decanter (a by-product of processing) by water-induced colloidal complexation performed at the base scale of 14 kg of decanter/batch was found to be economically sustainable considering the cost of manufacturing of USD 0.69 million, return on investment of 43.09%, and payback period values of just 2.32 years in a base-case scenario [65].

Parjikolaei et al. [145] evaluated the economic feasibility of using green and fossil-based organic solvents to extract astaxanthin from shrimp processing waste. Extraction utilizing hexane/isopropanol provided a higher yield of astaxanthin (26.4 kg/year) compared to SCE with 5% ethanol (12.8 kg/year). The cost of manufacturing using these methods was USD 0.6 and 0.82/mg of astaxanthin, respectively. On the other hand, the astaxanthin concentration obtained using sunflower oil and the methyl ester of sunflower oil was 2.5 and 153 ppm with a cost of manufacturing of USD 0.06 and 0.16/mg of astaxanthin, respectively. This final product obtained with the methyl ester of sunflower oil could be utilized directly in food and feed additives and thus has wider applications.

The techno-economic potential of AIBP valorization for polyphenols and carotenoids has not been widely investigated. However, the available studies suggest that it is an economically feasible process. In the future, more detailed studies on the development of modern low-cost extraction methods may further improve the economic feasibility of AIBP valorization.

## 5. Recovery of Oligosaccharides and Pectin

Pectin is a complex polysaccharide, mainly composed of homogalacturonan (HG) and rhamnogalacturonan (RG)-I, with lower amounts of RG-II and xylogalacturonan (XG) [146], widely found in the cell walls of plants, especially in fruits. It is commonly used in processed foods as an emulsifying, stabilizing, fat replacer, and thickening agent. The source and extraction procedure significantly impact the chemical composition of pectin [146]. In a comparative study, microwave-assisted extraction (MAE) provided a higher pectin yield of 29.17% from eggplant peel compared to eggplant calyx (18.36%) [146]. In addition, in pectin extracted from the peel, HG was the major fraction (58.6%), which provided higher water and holding capacity, emulsifying and foaming properties, and antioxidant activity compared to pectin extracted from calyx, which was high in RG-I (44.9%).

Citrus fruit peel is a rich source of pectin. The degree of esterification (DE) and the physiochemical properties of extracted pectin also widely depend on the extraction methods. For instance, extraction with acids decreases extraction yield and DE value, while the dynamic SWE technique yields pectin with relatively better solubility, lower molecular weight, and an amorphous structure [147]. In contrast, the pectin extracted via the conventional heat-based method with acid and sequential ultrasound-microwave assisted technique is naturally crystalline [147].

Hydrothermal treatment of avocado peel has been found effective in solubilizing oligosaccharides and antioxidant phenolics [109]. Treatment at 150 °C provided the highest recovery of total oligosaccharides, oligogalacturonides being the main component with the maximum content of 6.51 g/100 g of avocado peel. In contrast, treatment at 170 °C provided the significantly highest total flavonoid contents (TFC: 7.05 rutin eq./100 g), TPC (4.06 g gallic acid eq./100 g), and antioxidant activities.

Buriti (*Mauritia flexuosa* L. f.) fruit by-product flour investigated for its contents of dietary fiber and bioactive compounds revealed the presence of the highest amount of dietary fiber (88.91 g/100 g DW), non-extractable phenolics (proanthocyanidins; 5008.1 mg/100 g DW), and carotenoids (1186.7 mg/100 g DW) in unbalanced peel flour, compared to endocarp, bran, and pulp [43].

Solid-state fermentation utilizing food waste is environmentally friendly and economically viable to produce lignocellulolytic enzymes (e.g., cellulase, xylanase, and β-glucosidase). These enzymes can be utilized to disintegrate the cell wall of plant biomass and promote the release of cellular metabolites into the extraction solution. In one study, lignocellulolytic enzymes were produced by solid-state fermentation of agro-industrial wastes, including wineries, olive mills, and brewery wastes, and utilized for enhanced extraction of phenolic compounds. Among the various filamentous fungi tested, the highest xylanase and cellulase activities were achieved by *Aspergillus ibericus*, while *A. niger* CECT2088 was the best producer of β-glucosidase [148].

One pot protease treatment efficiently recovered high-quality oil, food-grade proteins, and fibers from pomegranate seed, a by-product of the pomegranate juice industry [110]. This study obtained the highest oil recovery of 22.9% and protein recovery of 13.2% when pomegranate seeds were incubated with 50 U/g protease at pH 7.2, 45 °C, and 14 h.

## 6. Microencapsulation

Bioactive compounds are sensitive to light, oxygen, moisture, pH, temperature, and metal-ions-mediated degradation. Several methods are employed to preserve the isolated bioactive compound, which include storage in amber or opaque containers, vacuum sealing or inert gas flushing to reduce oxygen exposure, use of synthetic antioxidants (e.g., BHT, propyl gallates) and metal chelators (e.g., EDTA, citric acid), and encapsulation techniques [149]. However, the use of chelators and synthetic antioxidants has several challenges, including legal constraints, functional drawbacks, and consumer concerns about their potential health risk [149]. Thus, encapsulation is the most promising and one of the most popular methods to protect bioactive compounds [150]. It also facilitates the application of hydrophobic compounds in aqueous solution. In addition, microcapsules can provide the controlled delivery of bioactive compounds to the target site, thereby improving their bioaccessibility and bioavailability [151]. Encapsulation technologies, such as freeze-drying, spray drying, coacervation, ionic gelation, and emulsification, are the most widely applied to food ingredients [150].

The microencapsulation of grape pomace phenolic extract by spray drying was optimized by Tsali and Goula [152] using the integrated mean-squared error optimal design method. The optimum values of encapsulation efficiency and yield of 92.49 and 37.28%, respectively, were predicated using the optimized process conditions of 1:1 maltodextrin/skim milk powder as encapsulating agent, 189 °C inlet air temperature, 8.8 wall-to-core-material ratio, and 65% (22.8 m^3^/h) drying air flow rate.

Andrade et al. [153] optimized the microencapsulation of phenolic-rich extract obtained from cashew apple waste extract using inulin (25%) and maltodextrin (75%), which showed good stability during 60 days of storage.

Red pepper waste extract rich in polyphenols (698.29 mg GAE/100 g) and carotenoids (63.97 mg β-carotene eq./100 g) encapsulated by freeze drying showed better quality in terms of solubility, moisture content, flowing, and color properties, compared to spray drying [154]. The freeze-dried capsules applied to yogurt retained 71.43% carotenoids and 123.73% polyphenol after 21 days of storage at 4 °C. In addition, encapsulated red pepper waste extract enhanced the sensory attributes of yogurt, including color and appearance, general acceptability, and flavor. Moreover, the fortification of yogurts with microencapsulated red pepper waste extract positively influenced maintainance of the initial count of lactic acid bacteria during storage.

The carotenoid-rich oil obtained from gac peel, encapsulated with a mixture of gum Arabic and whey protein concentrate using a spray dryer, showed better storage stability at 5 °C and 20 °C for 6 months compared to the infeed oil [155].

## 7. Applications of Bioactive Compounds Obtained from Food Waste and By-Products

Bioactive compounds obtained from AIBPs have great commercial applications (Table 3). Several bioactive compounds derived from food AIBPs are commercially available, underscoring their valorization’s importance. For instance, citrus peel extracts (rich in flavonoids), carrot pomace extract (rich in carotenoids), whey protein hydrolysates (rich in bioactive peptides), olive leaf extract (rich in polyphenols such as oleuropein), inulin (prebiotic fiber extracted from chicory roots), grape seed extract (rich in proanthocyanidins), and enzymes are but a few examples of the commercially available bioactive compounds derived from industrial by-products.

### 7.1. Food, Nutraceuticals, and Probiotics

Bioactive compounds obtained from AIBPs can be utilized for food fortification. Carotenoids extracted from carrot waste encapsulated utilizing the electrostatic extrusion technique using alginate as the carrier and incorporated in yogurt showed improved antioxidant activity with acceptable microbiological and physicochemical properties during the 28 d storage period [156]. This study provided evidence for utilizing the bioactive compounds extracted from food waste and for their appropriate utilization in developing fortified foods with improved nutritional properties.

Polyphenols and carotenoids are well known for their antioxidant potential. Enhanced intake of these compounds helps to reduce the oxidative stress in the body, thus minimizing the incidence of chronic diseases in the body [96,168,169]. A large cross-sectional study has shown a good association between lycopene consumption and lower risk of gestational diabetes mellitus (odds ratio: 0·50) in a Chinese population, particularly among primigravid women [170].

Methanolic extract, ethyl acetate fraction, and four major coumarins isolated from pomelo (*Citrus grandis* L.) showed anti-inflammatory properties in carrageenan-induced paw edema and xylene-induced ear edema in mice [171]. In this study, selected coumarins (e.g., auraptene) inhibited inflammatory cytokines, such as interleukin 1β (IL-1β), tumor necrosis factor α (TNF-α), and prostaglandin E2 (PGE2) induced by lipopolysaccharide (LPS) in RAW 264.7 cells. Interestingly, these coumarins-mediated inhibitory activities were comparable to dexamethasone, an anti-inflammatory drug. Similarly, unsaponifiable fractions (rich in β-Sitosterol, dammaradienol, cicloartenol, and α-tocopherol) isolated from grape seed oil have been shown to attenuate the oxidative and inflammatory responses in LPS-treated human primary monocytes by downregulating IL-1β, TNF-α, and IL-6 mRNA expression and secretion. Moreover, it showed significant reactive-oxygen-species-scavenging activities, significantly lowering nitrite levels and significantly reducing nitric oxide synthase 2 (Nos2) mRNA expression [172].

The pulp-enriched powder obtained from olive pomace solid fraction, rich in fatty acids, dietary fiber, and phenolics, showed that dietary fiber functions as a phenolics carrier and facilities their possible liberation in the stomach, allowing the recovery of a significant amount of phenolics, including tyrosol and its glucoside, as evident in the in vitro simulation of gastrointestinal digestion study [173]. Additionally, phenolics are retained in the colon, positively affecting gut health. In addition, dietary fiber demonstrated a positive interaction with lipids, facilitating the absorption of anti-thrombogenic unsaturated fatty acids and decreasing the bioaccessibility of pro-thrombogenic saturated fatty acids.

Non-digestible carbohydrates and non-carbohydrate substances function as prebiotics. The resident microbiota ferments these in the colon to produce short-chain fatty acids (SCFAs), including butyrate, acetate, and propionate [174]. SCFAs are well known to promote the growth of specific health-beneficial microbiota, such as Lactobacillus ssp. and Bifidobacterium spp. In addition, SCFAs are directly or indirectly involved in the healthy gut barrier, anti-inflammatory properties, metabolic regulations, and maintaining the brain–gut axis and good mental health [174]. Interestingly, these prebiotics can be derived from low-cost and widely available agro-industrial by-products, such as fruit pomace, cereal and grain by-products, and molasses [22].

Most valorization studies have investigated the recovery and purification of individual bioactive compounds. However, fruit by-products can also be incorporated directly into existing food products. Blueberry (pomace) and persimmon by-products (peels and chalices) rich in polyphenols and carotenoids were freeze-dried, powdered, and utilized as a carotenoid-, anthocyanin-, and dietary-fiber-rich high-value ingredient in food formulation [175]. The colonic fermentation of this ingredient leads to the growth of health-beneficial *Ruminococcaceae* genus and Lactobacillus, with suppression of harmful Streptococcus. Additionally, in this study, the health-beneficial Actinobacteria genera (*Bifidobacterium* and *Collinsella*), *Ruminococcaceae*, and *Akkermansia* were positively correlated with polyphenols. Moreover, *Faecalibacterium* and *Bifidobacterium* correlated positively with fiber contents.

### 7.2. Stabilizer in Vegetable Oil

Vegetable oils, especially rich in PUFAs, are susceptible to oxidation, resulting in shelf-life reduction by forming off-flavors and toxic compounds, unpleasant odors, reduction in nutritional value, and overall quality deterioration. Pomegranate peel extract (100–300 ppm), rich in phenolic compounds, effectively minimizes oxidative changes during accelerated soybean and mustard oil storage at 60 °C for 24 days [176]. In particular, in this study, pomegranate peel extract nanoencapsulated with maltodextrin and whey protein isolate showed better protection than the synthetic antioxidant (butylated hydroxytoluene, BHT) and unencapsulated extract. The nanoencapsulated extracts were more efficient in preventing oxidation, probably due to the controlled release of phenolic compounds. In another study, the rich phenolic compounds found in pomegranate peel extract (0.05%) alone or in combination with synthetic antioxidant (0.01% BHT) provided significantly better stability of pomegranate seed oil during storage for 12 days at 65 °C, compared to synthetic antioxidant [177]. Moreover, in this study, pomegranate peel extract was shown to be the most appropriate antioxidant to prevent the second stage of oxidation, which is characterized by the decomposition of hydroperoxides to chain-shortened alcohols and aldehydes, causing a rancid smell. Moreover, the combination of pomegranate peel extract and BHT showed better stability in the primary oxidation process, characterized by hydroperoxide formation.

Crude extract provides better protection for vegetable oil than purified compounds. For instance, 200–1000 ppm mango peel extract, rich in β-carotene, prevented sunflower oil lipid oxidation. In contrast, no protective effects were observed in this study using pure β-carotene at an equivalent concentration [54], suggesting that other constituents in the extract protect from lipid oxidation.

### 7.3. Biodegradable Packaging Materials

The polysaccharides, proteins, and lipids extracted from food and by-product waste can be utilized in manufacturing biodegradable packaging materials [178,179,180]. The most used polysaccharides include alginate, chitosan, carrageenan, starch, cellulose derivatives, pectin, and pullulan. Similarly, proteins, such as corn zein, soy protein, gelatin, wheat gluten, and lipids, including triglycerides and vegetable oils, are widely used. Moreover, fruit and vegetable waste (e.g., citrus peel) powder can be directly utilized to produce active packaging films without isolating polymer or other active compounds [179]. Using cheaper, underutilized food-processing by-products is a promising strategy for producing biodegradable packaging material. Most importantly, using natural biodegradable materials for packaging can offer several environmental, economic, and functional benefits (e.g., strength, flexibility, and antimicrobial effects).

The polyphenol extracts of Ilex paraguariensis (a by-product of yerba mate) utilized in producing bioactive cornstarch films showed enhanced plasticizer properties with improved antioxidant, mechanical, and thermal properties [181]. The physicochemical properties suggested that the prepared film may be utilized to prevent light-triggered oxidative deterioration and maintain the freshness of products. Similarly, fish gelatin film-forming solution incorporated with pomegranate (*Punica granatum* L.) peel powder improved the water vapor permeability, tensile strengths, and antioxidant and antimicrobial (especially against Staphylococcus aureus) properties of the fish gelatin film [182]. These results showed that bioactive compounds found in food waste have great potential to be used as an active ingredient in biodegradable films, which can be utilized to maintain the quality and enhance the shelf life of food products.

Cellulose nanofibers obtained from a vegetable source (e.g., banana peel) have shown promise for use in biodegradable films. The alkaline treatment (5% KOH, *w*/*v*), followed by bleaching (1% NaCIO_2_, at 70 °C for 1 h), acid hydrolysis (1–10% H_2_SO_4_ at 80 °C for 1 h), and mechanical treatment (i.e., high-pressure homogenization) can produce nanometric cellulose fibers from the banana peel [183]. These cellulose fibers were non-cytotoxic to Caco-2 cells up to 500 mg/mL, suggesting no adverse impact on human health.

#### Active and Intelligent Packaging

Antioxidant and antimicrobial compounds from AIBPs can be utilized efficiently to fabricate active and intelligent packaging. The antioxidant and antimicrobial compounds (e.g., polyphenols) are incorporated in the fabrication of active packaging film. Antioxidants help minimize the lipid oxidation process by detoxifying the free radicals. In contrast, antimicrobial compounds inhibit or delay the growth of harmful microbes. These properties assist in reducing food spoilage, prolonging shelf life, and maintaining the sensory quality of perishable food, such as seafood and meat products [158,184].

Similarly, the pH-sensitive properties of bioactive compounds (e.g., anthocyanins and betalians) are utilized in intelligent packaging to monitor food freshness in real time [158]. These bioactive compounds act as natural pH indicators, changing color in response to variations in food spoilage conditions, such as microbial activity, pH shifts, or gas emissions (e.g., ammonia, biogenic amines, and CO_2_) [185].

Conventionally, paraffin-wax-based edible coating is applied to increase the fruits’ shelf life. Conversely, due to the harmful effects of chemical-derived waxes, there has been considerable research and progress in discovering natural and safe ingredients for coating foods. These paraffin-wax-based edible coatings can be replaced by edible film and coatings (formed on the food product by dipping or spraying material) fabricated from the AFWB [186]. Edible film enhances the shelf life by creating a semi-permeable protective covering around the fruit and vegetable surface, thus minimizing the respiration, phenol oxidation, and evapotranspiration rate and modifying the gaseous environment (e.g., O_2_ and CO_2_) [157]. In addition, the fruit coating fills up the tiny cracks on the fruit’s pericarp, causing stomata and lenticels to close and postponing the onset of enzymatic reactions and physiological disorders [157].

AIBP, rich in dietary fiber and bioactive compounds, can be utilized to improve the nutritional quality of baked goods [187]. Since baked goods have a high glycemic index due to cereal-based starch and carbohydrates, dietary fibers, such as pectin, can help slow digestion. However, adding fruit and vegetable by-products has shown a loss in food acceptability and sensory quality, mainly due to changes in flavor and appearance [187]. Thus, intensive efforts are required to find the optimal composition and physical properties of fruit and vegetable by-products that attenuate the negative sensorial properties while maximizing the nutritional benefits. Antioxidant chitosan–banana peel extract composite film showed good antioxidant activity and enhanced preservation of apples during ambient temperature storage by minimizing the respiration rate, weight loss, firmness, titratable acidity, and ascorbic acid contents compared to the uncoated group [188].

Bioactive edible packaging films obtained by incorporating 10% tea polyphenols into the pomelo peel flour showed improved antimicrobial and antioxidant activity of the films [189]. Moreover, adding tea polyphenols decreased the moisture content, transmittance, and elongation at break. The possible food packaging application evaluated using soybean oil (filled in a glass bottle and covered with prepared film) in an oven at 50 °C for 30 days showed a significant delay in the oil oxidation. In another study, an antioxidant-active food packaging film prepared utilizing natural bioactive extract and cellulose nanofiber from orange peel waste showed increased UV-light barrier properties [161].

## 8. Circular Bio-Economy Approach

Reduced underutilization of processing by-products, increased reuse, and less strain on natural resources and systems are the goals of the circular economy [190]. It is a fast-growing sector contributing to increased materials and energy production with minimal environmental impact [191]. A biorefinery and circular economy approach should be followed to utilize AIBP effectively. This approach includes sequential extraction or cascade extraction, where bioactive compounds of high economic value such as polyphenols and carotenoids are extracted first; the generated waste can be further subjected to processes including, but not limited to, biofuel (e.g., biodiesel, biomethane, biohydrogen) and bioplastic (e.g., polyhydroxyalkanoates, cellulose regenerates, and polybutylene terephthalate) production [191]. For instance, citrus processing waste can be subjected to essential oil extraction using distillation followed by dilute acid hydrolysis to isolate pectin and bioethanol with yeast (e.g., *Pichia kudriavzevii* KVMP10). The remaining solid residues obtained after acid hydrolysis can be anaerobically digested to procure biomethane [192]. In a biorefinery approach, Espinosa et al. [161] utilized orange peel waste to prepare polyphenolic-rich bioactive extract and cellulose nanofibers, which together were incorporated into a poly(vinyl alcohol)-based film with antioxidants and increased the UV-light barrier properties.

Olive processing waste is a rich source of polyphenols; thus, in addition to the polyphenols’ extraction, the residual biomass can be used in the production of economically important nanocelluloses, cellulose nanofibrils, cellulose nanocrystals, and lignin-containing cellulose nanofibrils [193].

Aquaculture waste, which mainly includes by-products from fish, shellfish (e.g., mollusks and crustaceans), and seaweeds, can be utilized to produce high-value compounds, including proteins, collagen, gelatine, chitosan, carotenoids (astaxanthin, β-carotene, and fucoxanthin), and n3 FUFAs [190,194]. McElroy [195] developed an integrated biorefinery approach to valorize seaweed *Saccharina biomas* to isolate fucoxanthin utilizing SCE, followed by mannitol (4.15% yield (*w*/*w*)) extraction using an MAE, and finally isolated fucoidans and alginates from the residual biomass. This study obtained the highest value of 1.26 mg/g of carotenoids at 50 °C and 20 MPa pressure.

## 9. Challenges in AIBPs Valorization

Significant advancement has been made in the valorization of AIBPs [9,18,20,21,22,76,196]. However, numerous challenges still exist; these challenges and their possible solutions are summarized in Table 4.

## 10. Safety Hazards Related to AIBPs Valorization

AIBPs are a potential source of industrially vital compounds. However, valorization of such waste presents several challenges, including the presence of pesticides, mycotoxins (e.g., aflatoxins, ochratoxin A, deoxynivalenol, and zearalenone), foodborne pathogens (e.g., *Salmonella* spp. and *E. coli*) and molds, heavy metals (e.g., mercury, lead, cadmium, and arsenic), and biogenic amines [23,201].

Most pesticides used in crops are hydrophobic and are easily solubilized in hexane and acetonitrile [202]. Thus, using these solvents to extract bioactive compounds increases the risk of pesticide contamination. One study has shown 28–60% degradation of the pesticide residues present in the food matrix by thermal processing at 75 °C to 85 °C, 35 min. In particular, in this study, difenoconazole showed the highest degradation rates, probably associated with its thermal instability [203].

Another major concerns is the presence of mycotoxins, especially aflatoxins, ochratoxin A, deoxynivalenol, and zearalenone in agro-food by-products [23]. The most effective way to eliminate the presence of mycotoxins and ensure food supplement safety is by preventing fungal growth by implementing good agricultural practices in crop production, harvesting, and storage, which may include using bio-safe post-harvest detoxifying methods, storing the food commodities at suitable storage conditions with appropriate environmental control, and following strict quality assurance procedures [204]. Additionally, utilizing absorbent materials during extraction can help to decontaminate the plant material from mycotoxin [205]. A variety of adsorbents, including activated charcoal, bentonite, yeast cell wall, and hydrated sodium calcium aluminosilicate, have been studied for the adsorptive removal of mycotoxin [205].

## 11. Conclusions and Future Perspective

The valorization of agro-industrial by-products (AIBPs) for recovering economically viable bioactive compounds has not yet been fully explored. Currently, most such attempts are concentrated at the lab scale, and actual application at the industrial scale is not applied. Moreover, a comprehensive techno-economic assessment of the extraction of bioactive compounds from food wastes and by-products remains to be performed. The cost may significantly differ with changes in the food wastes and by-products, extraction method, and target compound(s). Thus, detailed investigations are required in these fields.

Natural deep eutectic solvents (NADES) are potentially emerging green solvents that offer several advantages over conventional organic solvents. Nevertheless, there is currently little to no research on the direct use of NADES extracts in food products. Moreover, further research is needed to optimize the efficiency and yield of bioactive compounds from food waste and by-products.

The majority of research has focused on the total phenol content of samples, with little consideration given to non-extractable polyphenols (NEPs), a significant class of polyphenols, underestimating the total phenolic contents of the sample. A suitable approach should be applied for the correct estimation, including sequential extraction with alkaline, acid, or enzymatic hydrolysis to release the NEPs from the sample matrix.

The use of active, intelligent, and edible film can solve several issues related to toxicity and the environment. However, the costs of such films are very high compared to conventional materials. Using inexpensive and underutilized food wastes and by-products can help reduce production costs. Most research on developing edible film is limited to a laboratory scale. Several variables, including the practicality, cost analysis, and storage stability of active and intelligent packaging, require additional evaluation to make it more industry adaptable. Additionally, investigations on upscaling manufacturing at a commercial scale with economic viability are required. Furthermore, detailed in vivo studies are required to determine the cytotoxicity of various polymeric matrixes used for edible films.

## Figures and Tables

**Table 1:** Bioactive composition of agro-industrial by-products (AIBPs).

Agro-Food Industry	By-Product/Waste	Bioactive Compounds	Reference
Shrimp processing	Head and carapace residues (38.1–45.4%)	LC-n3-PUFAs including docosahexaenoic acid (DHA), eicosapentaenoic acid (EPA), and astaxanthin (31–84 µg/g FW), α-tocopherol (32.0–35.3 µg/g FW), and sterols.	[6,25]
Sardine (*Sardina pilchardus*) canning	Viscera, spines, and heads	LC-n3-PUFAs, including EPA and DHA, and amino acids	[26]
Olive processing	Olive pomace	α-Tocopherol (2.63 mg/100 g), oleic acid (75% of total fatty acids), hydroxytyrosol (83.6 mg/100 g), comselogoside, and triterpenic acids (maslinic acid)	[27,28]
Olive processing	Leaves (10% of the weight of the olives delivered to the mill)	Triterpenes, including oleuropein, α-amyrin, oleanolic acid, and maslinic acid	[29]
Potato processing	Peel (6–10%)	Polyphenols (15.8–32.2% DW) and starch (52.1% DW)	[30]
Tomato processing	Pomace, seed, and skin	Tocopherols, polyphenols (mainly ellagic and chlorogenic acids, rutin, and myricetin), terpenes, minerals, and sterols; the peel mainly contains high amounts of lycopene	[10,31,32,33]
Pineapple processing	Crown, core, peels, and fruit trimmings (55–70%)	Bromelain enzyme, caffeic acid-O-hexoside, and apigenin 6,8-C-diglucoside	[34,35]
Broad beans (*Vicia faba*) processing	Green pods (after seed removal)	Dietary fiber (57.46% DW), carbohydrate (18.93% DW), protein (13.81% DW), linoleic acid (39.74% of total fatty acids), carotenoids (7.3 µg/g FW), minerals (mainly K), and polyphenols	[36]
Avocado (*Persea americana* Mill.) processing	Peel and kernel (30%)	Polyphenols (mainly epicatechin), chlorogenic acid derivatives, and oligosaccharides	[37]
Chestnut peeling/processing	Chestnut shells and inner chestnut shells	Gallic acid, protocatechuic acid, and condensed tannins	[38]
Ginkgo biloba fruit processing	Seed exocarp	Polysaccharides, flavonoids, terpene trilactones, and ginkgolic acids	[39]
Pomegranate (*Punica granatum* L.) juice production	Peel (30–40%)	Tannins (pomegranatins A–C, gemin D, casuariin, punicacortein D, punicacortein C, pedunculagin and granatin A), phenolic acids (caffeic, chlorogenic, syringic, ferulic, and gallic acid), and flavonoids (anthocyanins, catechin, quercetin, and epicatechin)	[40,41]
Citrus juice processing	Pomace (seeds, peel, and pulp; 50–60% of fruits)	Polyunsaturated fatty acids, pectin, organic acid, dietary fiber, limonoids, carotenoids, vitamins, and polyphenols, including apigenin-7-O-glucoside, quercetagetin, hesperetin-7-O-rutinoside, peonidin, cyanidin, quercetin, naringenin, and cyanidin-3,5-di-O-glucoside	[42]
Buriti (*Mauritia flexuosa* L. f.) fruit oil	Peels, endocarp, and pulp bran	Dietary fiber (88.91 g/100 g DW), non-extractable phenolics (proanthocyanidins; 5008.1 mg/100 g DW), and carotenoids (1186.7 mg/100 g DW) in unbalanced peel flour	[43]
Walnut (*Juglans regia* L.) processing	Walnut fructus (the dry wooden diaphragm inside walnuts)	Total dietary polyphenols of 2878.1–6183.5 µg/g, consisting mainly gallic acid (89.8–216.5 µg/g), protocatehuic acid (44.3–154.1 µg/g), (+)-catechin (251.7–693.3 µg/g), (−)-epicatechin gallate (22.3–194.8 µg/g), taxifolin (34.0–153.3 µg/g), ellagic acid (518.3–1733.7 µg/g), isoquercitrin (32.3–116.7 µg/g), taxifolin-3-O-arabinofuranoside (519.6–2181.9 µg/g), and quercitrin (145.4–983.6 µg/g)	[44]
Winemaking industry	Grape skin (50%), seeds (25%), and pomace	(+)-catechin, (−)-epicatechin), epigallocatechin, anthocyanins, procyanidins dimers, kaempferol, quercetin, vanillic, syringic, gallic, protocatechuic, ellagic acids, and stilbenes (resveratrol)	[45,46]
Winemaking industry	Wine lees (primarily dead yeast cells)	Caftaric acid (cis and trans) coutaric acids, caffeic acid, p-coumaric acids, hydroxybenzoic acids (gallic and 2,5 dihydroxybenzoic acids), astilbin (flavanone glycoside), and catechin (flavanol)	[47]
Cocoa (*Theobroma cacao* L.)	Cocoa pod husk (70–75% dry weight of whole fruit)	Organic acids, fatty acids, procyanidins, amino acids, alkaloids, pectin, minerals (mainly potassium, 2.8–3.8% *w*/*w*), fiber (including cellulose, hemicellulose, pectin, and lignin), and polyphenols	[48,49]
Cocoa/ chocolate industry	Cocoa shells (byproduct of roasting the beans)	Flavanols (catechin and epicatechin), methylxanthines (theobromine and caffeine), fatty acids (964 mg/g extract), and fibres	[50]
Mushroom production	Spent mycelium substrate	Polysaccharides (α-D-glucans, β-D-glucans), chitin, proteins, polyphenols, fatty acids, vitamins, and minerals	[51]
Red pitaya (*Hylocereus costaricensis*) processing	Peel	Oxalic acid (1.68 g/100 g DW), malic acid (1.7 g/100 g DW), betacyanin (betanin, isobetanin, phyllocactin, phyllocactin, and isophyllocactin), and γ-tocopherol	[52]
Acerola (*Malpighia emarginata* DC.)	Seeds and pomace (20%)	Quercetin (quercetin O-rhamnoside; quercetin pentosyl-O-hexoside), kaempferol (kaempferol O-rhamnoside), and ascorbic acid	[53]
Mango processing	Peels (15–20%) and stone (20–45%)	β-carotene, chlorophylls, 13-cis -β-carotene, xanthophylls, lupeol, α-amyrin, tocopherols, dehydroascorbic acid, luteolin-7-O-glucoside, rutin, mangiferin, quercetin 3-O-galactoside, and pectin	[54,55]
Onion (*Allium cepa* L.) processing	Peel	Quercetin, myricetin, kaempferol, quercetin 4′-O-glucoside, cyanidin 3-(6″-malonylglucoside), and cyanidin 3-O-glucoside	[56,57]
Kiwifruit farming and processing	Peel, pomace, and undersized fruits	Epicatechin (2.295 mg/g DW), quercetin (0.023 mg/g DW), (+)-catechin, protocatechuic acid, p-coumaric acid, caffeic acid, and chlorogenic acid	[58]
Jackfruit (*Artocarpus* *heterophyllus* Lam.) processing	Peel (55–62%)	Ascorbic acid, quinic acid, shikimic acid, catechin, gallic acid, and chlorogenic acid	[59,60]
Papaya (*Carica papaya* L.) processing	Seeds (15–20%) and peel	Pectin and phenolic acids including 3,4 dihydroxybenzoic acid, 4-hydroxymethylbenzoic acid, ferulic acid, chlorogenic acid, gallic acid, methoxyphenylacetic acid, salicylic acid, vanillic acid, myricetin, and resveratrol	[61,62]
Passion fruit (*Passiflora edulis* sp.) processing	Rind (60% of the total fruit mass)	Flavonoids, including C-glycosyl isoorentin, isovitexin vitexin, and vicenin	[63]
*Capsicum annuum* bell pepper farming	Undersized and damaged bell peppers	Carotenoids, including violaxanthin, lutein, β-carotene, and zeaxanthin; and phenolic acids, including feruloyl-hexoside, sinapic acid-O-hexoside, and galloyl-1,4-galactarolactone	[64]
Guava (*Psidium guajaya*) fruit processing	Peels, seeds, residual pulp, and decanter	Lycopene, pectin, phenolic acids (vanillin and vanillic acid), and fatty acids (linoleic acid, palmitic acid, and oleic acid)	[65,66]
Marigold (*Tagetes* spp. L.) flowers; ceremonial, decorative, or recreational activities	Flower petals	Carotenoids (25.62–2723.11 µg/g FW; predominantly lutein-diesters), α-tocopherol (167.91–338.50 µg/g FW), phytosterols ( β-sitosterol; 127.08–191.99 µg/g FW), and fatty acids	[67]

Abbreviations: DW, dry weight; LC-n3-PUFAs, omega-3 (n3) long-chain (LC) polyunsaturated fatty acids (PUFAs).

**Table 3:** Commercial applications of bioactive compounds extracted from agro-industrial by-products (AIBPs).

Bioactive	Bioactive Properties	Source	Applications	Reference
Astaxanthin	Antioxidant Natural coloration	Crustacean processing waste	Animal feed Food Dietary supplements Pharmaceuticals Cosmetics	[6]
Protein	Nutritional (sources of several essential amino acids) Gelling Emulsifying and foaming	Whey from dairy processing, collagen and gelatin from meat and seafood processing, zein from corn processing, and rice bran from rice mill	Revalued Food Bioplastics Edible films and coating Water purification (functional superabsorbent) Renewable energy	[20]
Bromelain enzyme	Immunomodulatory Protease activity	Pineapple waste	Nutraceutical Cosmetics Meat tenderization Anti-browning agent in fruit processing Baking industry (enhance dough relaxation and permits the dough to rise evenly) Protease in detergents, textile manufacturing, and leather processing	[34]
Carotenoids	Antioxidant Natural colorant		Yogurt fortification	[156]
Starch	Hydrocolloid film forming		Edible Packaging film and coating	[157]
Anthocyanins	pH-sensitive Antioxidant		Fabrication of intelligent packaging film Food fortification Food preservation	[158]
Polyphenols	Antioxidant Antimicrobial	Rice straw, thinned fruits	Fabrication of active packaging film Antioxidants for functional foods Food preservatives and stabilizers	[158,159,160]
Polyphenolic extract and cellulose nanofiber	Antioxidant UV-light blocking	Orange peel	Active packaging film	[161]
Cellulose nanofibers	No cytotoxicity to COS-7 cells Absorb casein micelles within hydrogen bonds to form a gel-like structure	Brown algae	Milk thickeners	[162]
Cellulose and cellulose nanocrystals	Film forming	Rice straw, pistachio shells, peanut shells, orange peel	Sustainable bioplastic Edible film and coating Stabilizer for pickering emulsion	[159,163,164,165]
Starch, calcium (Ca), and silicon (Si)	Film forming and nanocrystalline	Cassava (Manihot esculenta) peel and bagasse	Biodegradable food packaging	[166]
Chitin	Antimicrobial Moisture barrier	Mushroom waste	Biodegradable food packaging film	[167]

Abbreviation: COS-7, African green monkey kidney fibroblast cells.

**Table 4:** Challenges associated with AIBPs’ valorization and possible solutions to mitigate such challenges.

Challenges	Possible Solution	Reference
AIBPs are highly sensitive to microbial spoilage due to their high moisture content.	Dehydration of AIBPs can help significantly reduce the activity of enzymes and microbes. Moreover, it facilitates ease of handling and distribution of the by-product due to the substantial reduction in the weight and volume of biomass.	[98]
The use of organic solvents for the extraction creates new environmental issues and also poses the risk of solvent residues in the extract.	Green-solvent-assisted extraction utilizing supercritical CO_2_, terpenes (e.g., limonene), and deep eutectic solvents (DESs) can help to minimize the risk of solvent residues in the extract.	[132,133,135]
The isolated bioactive compounds and extract are sensitive to light, oxygen, moisture, pH, temperature, and metal-ions-mediated degradation.	The encapsulation of isolated bioactive compounds and extracts can significantly improve storage stability. In addition, it can help improve bioaccessibility and bioavailability.	[149,151]
Extraction and purification of individual compounds are usually associated with high costs (low concentrations in a complex matrix); thus, the economic aspects are still critical.	Direct applications of AIBPs in food products. For instance, seeds obtained as bioproduct quince (*Cydonia oblonga* Mill.) fruit processing can be directly utilized in food. Using bioactive-rich extracts directly, instead of first purifying bioactive compounds, can significantly reduce production costs. Moreover, the combined action of multiple bioactive compounds in an extract can provide synergistic effects. More investigations are required to develop economically feasible extraction methods.	[197,198]
The extraction of small amounts of bioactive material does not eliminate the bulk mass of food waste by-products.	A biorefinery and a circular bioeconomy approach can be used, including sequential extraction of primary and secondary metabolites, followed by using remaining solid residues for biofuel production.	[65,143,190,191]
The extraction yields are not optimum.	The section on appropriate extraction methods and optimization of extraction parameters is crucial for achieving the optimum yield. Modern techniques need to be introduced to improve the recovery of bioactive compounds.	[199,200]

## Data Availability

Data are contained within the article.
